# Random migration of induced pluripotent stem cell-derived human gastrulation-stage mesendoderm

**DOI:** 10.1371/journal.pone.0201960

**Published:** 2018-09-10

**Authors:** Yuta Yamamoto, Shota Miyazaki, Kenshiro Maruyama, Ryo Kobayashi, Minh Nguyen Tuyet Le, Ayumu Kano, Akiko Kondow, Shuji Fujii, Kiyoshi Ohnuma

**Affiliations:** 1 Department of Bioengineering, Nagaoka University of Technology, Nagaoka, NIIGATA, Japan; 2 Department of Science of Technology Innovation, Nagaoka University of Technology, Nagaoka, NIIGATA, Japan; 3 Division of Biomedical Polymer Science, Institute for Comprehensive Medical Science, Fujita Health University, Toyoake, Aichi, Japan; 4 Department of Materials Science and Technology, Nagaoka University of Technology, Nagaoka, NIIGATA, Japan; Okayama University, JAPAN

## Abstract

Gastrulation is the initial systematic deformation of the embryo to form germ layers, which is characterized by the placement of appropriate cells in their destined locations. Thus, gastrulation, which occurs at the beginning of the second month of pregnancy, is a critical stage in human body formation. Although histological analyses indicate that human gastrulation is similar to that of other amniotes (birds and mammals), much of human gastrulation dynamics remain unresolved due to ethical and technical limitations. We used human induced pluripotent stem cells (hiPSCs) to study the migration of mesendodermal cells through the primitive streak to form discoidal germ layers during gastrulation. Immunostaining results showed that hiPSCs differentiated into mesendodermal cells and that epithelial–mesenchymal transition occurred through the activation of the Activin/Nodal and Wnt/beta-catenin pathways. Single-cell time-lapse imaging of cells adhered to cover glass showed that mesendodermal differentiation resulted in the dissociation of cells and an increase in their migration speed, thus confirming the occurrence of epithelial–mesenchymal transition. These results suggest that mesendodermal cells derived from hiPSCs may be used as a model system for studying migration during human gastrulation *in vitro*. Using random walk analysis, we found that random migration occurred for both undifferentiated hiPSCs and differentiated mesendodermal cells. Two-dimensional random walk simulation showed that homogeneous dissociation of particles may form a discoidal layer, suggesting that random migration might be suitable to effectively disperse cells homogeneously from the primitive streak to form discoidal germ layers during human gastrulation.

## Introduction

**Fig 1:**
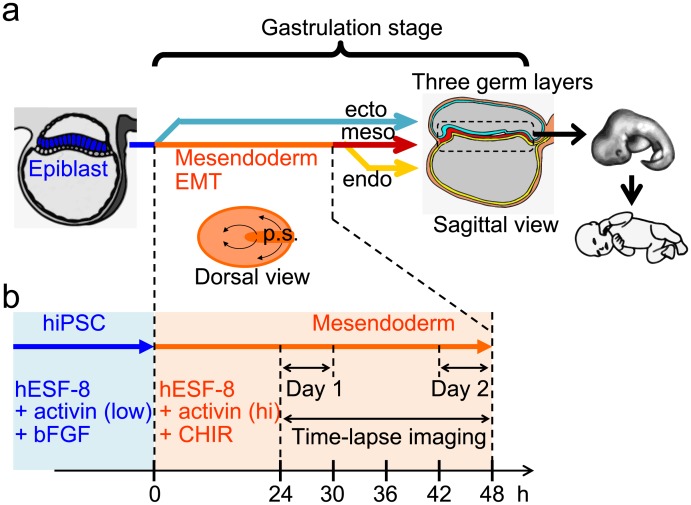
Schematics of human gastrulation *in vivo* and time-lapse imaging of hiPSCs *in vitro*. a: Schematics of human gastrulation *in vivo*; ecto: ectoderm (that forms neurons), meso: mesoderm (that forms the muscle, blood, and bone), and endo: endoderm (that forms the intestine). Dorsal view: arrows indicate cell movement and p.s. indicates primitive streak. b: Chronological diagram for time-lapse imaging of hiPSC-derived mesendodermal cells mimicking the mesendoderm during human gastrulation. We defined 24–30 h and 42–48 h of mesendodermal differentiation as day 1 and day 2, respectively.

Gastrulation, which is critical to human body formation, is the initial dynamic deformation of the embryo and occurs at the beginning of the second month of pregnancy, which is 2–3 weeks after fertilization (Fig 1a) [1–3]. Gastrulation in humans is similar to that in other amniotes (birds and mammals), especially chickens, which form a laminar disc-shaped embryo called an epiblast before gastrulation [2]. During amniote gastrulation, the epiblast, which is comprised of epithelial cells, differentiates into the ectoderm, which forms the neural system, and the mesendoderm, comprised of mesenchymal cells that form the muscles, heart, bone, and the digestive tract. The epithelial to mesenchymal transition (EMT), which occurs during mesendodermal differentiation, endows cells with migratory and invasive properties [2, 4]. Mesendodermal cells migrate to their appropriate locations through the primitive streak to form the discoidal mesoderm and endoderm layers. The three germ layers, ectoderm, mesoderm, and endoderm, derived from the epiblast, in turn, form the entire body (Fig 1a). Therefore, gastrulation is closely associated with birth defects [3]. Quantitative measurement of the dynamics of human gastrulation is important in developmental biology as well as in medical services. Although histology as well as genetic regulation of early human embryogenesis have been studied [5, 6], cellular dynamics of early human embryogenesis is not well understood largely due to ethical and technical limitations.

Human induced pluripotent stem cells (hiPSCs) and human embryonic stem cells (hESCs) share similar properties such as pluripotency and infinite self-renewal capacity, and therefore provide good models for studying human gastrulation dynamics *in vitro* (Fig 1a) [7–9]. The hiPSCs correspond to epiblasts, differentiate into mesendodermal cells, and undergo EMT within a few days through the activation of the Activin/Nodal and Wnt/β-catenin signaling pathways [9–12]. In addition, hiPSC differentiation can easily be regulated using a defined culture medium [13–15]. Moreover, the dynamics of each hiPSC can easily be determined by examining single-cell monolayer cultures of hiPSCs under a microscope [16–18]. Despite these advantages, the use of hiPSCs in human gastrulation dynamics studies is still limited [19]. Furthermore, to our knowledge, no studies have been conducted on the randomness of human mesendodermal cells.

In the present study, we analyzed the activity of mesendodermal cells derived from hiPSCs to determine the dynamics of mesendodermal cells during human gastrulation. Time-lapse imaging was performed to analyze the speed and randomness of cell migration via the tracking of single-cell movement.

## Materials and methods

### Culturing of hiPSCs

The hiPSC line 201B7 [8] was obtained from Riken BRC Cell Bank (Tsukuba, Ibaraki, Japan) through the National Bio-Resource Project for the Ministry of Education, Culture, Sports, Science and Technology, Japan. These hiPSCs were cultured as described previously [20–22]. Briefly, the cells were maintained in undifferentiation-maintaining medium ESF9a containing hESF-8 medium (S1 Table) supplemented with 10 ng/mL basic fibroblast growth factor (bFGF) and 2 ng/mL human recombinant activin A on 2 μg/mL fibronectin-coated dishes. For inducing differentiation, the culture medium was replaced with mesendoderm induction medium containing ESF-8 medium, 10 ng/mL activin A, and 12 μM CHIR99021 (CHIR).

### Close-packed cell density

Cells were plated at a density of 4 × 10^5^ cells/cm^2^ and harvested for cell counts 1 to 3 days later. Close-packed cell density was determined from the saturated cell number (4.5 × 10^^5^ cell/cm).

### Immunocytochemical analysis

The hiPSCs were fixed with 4% paraformaldehyde for 20 min. The cells were permeabilized and blocked with PBS containing 0.2% Triton X-100 and 10 mg/mL bovine serum albumin for 60 min. Primary and secondary antibody information is listed (S2 Table). Nuclei were stained with 0.4 μM DAPI (Wako Pure Chemical Inc.). Micrographs were obtained using a BZ-8100 microscope (Keyence, Osaka, Japan).

### Time-lapse imaging

Glass-based dishes (3960–035, Iwaki, Japan) were prepared by wiping the surface with ethanol and coating with polydimethylsiloxane (PDMS; Sylgard 184 Silicone Elastomer Kit; Dow Corning Toray Co., Ltd., Tokyo, Japan) using a spin coater at 1000 rpm for 60 s and then at 3000 rpm for 120 s (MSA-100; Mikasa Co., Ltd., Tokyo, Japan), followed by heat curing. Next, a heat-cured PDMS flame with two holes (diameter, 10 mm) was used to bond the glass-based dishes by using O_2_ plasma (SEDE-P; Meiwaforsis, Tokyo, Japan) to make two-well dishes. The bottom of the two-well dishes was coated with 0.5 μg/cm^2^ vitronectin (2349-VN-100; R&D Systems, MN, USA) and left overnight at 37 °C. The hiPSCs were harvested and dissociated into single cells by incubation with 0.02% (w/w) ethylenediaminetetraacetic acid (EDTA) in phosphate buffer solution (PBS) for 10 min and were suspended in either undifferentiation-maintaining medium or mesendoderm induction medium containing 5 μM ROCK inhibitor (Y-27632; Wako Pure Chemical Industries, Ltd., Osaka Japan). The cells were plated inside the PDMS frame (cell density, 1 × 10^4^ cells/cm^2^) and cultured for 1 day to promote their adherence to the dish. Prior to performing time-lapse imaging, the cells were stained with 100 ng/mL Hoechst 33342 (DOTITE3 46–07951; Wako Pure Chemical Inc.), a nuclear dye, for 30 min (Fig 1b).

The two-well dishes were placed in an observation chamber supplied with humidified 5% CO_2_ and 95% air. Next, the dishes were examined using an inverted microscope Eclipse Ti-E (Nikon Instech Co., Ltd., Tokyo, Japan) equipped with a motor-driven X/Y stage (BIOS-105T, SIGMAKOKI CO., LTD, Tokyo, Japan), a thermal insulation system (temperature, 37 °C; Nikon Instech Co., Ltd), and a CMOS camera (pco.edge 4.2, PCO AG, Germany). The microscope system was controlled using μManager software (Open Imaging, Inc. https://open-imaging.com/). Movement of Hoechst 33342-stained nuclei was tracked using ImageJ software (NIH, MD USA) with the Fiji and TrackMate plugin. In regard to cell division, one sister cell was tracked while the other was tracked as a different cell that started at the point of division. Cells that moved out of the field of view were not analyzed. Time-lapse images were taken at 15 min time intervals and a total of 60 undifferentiated and differentiated cells on day 1 and 40 differentiated cells on day 2 were analyzed. Calculations were performed via importing cell coordinates into Microsoft Excel.

### Random motion analysis

Mean square displacement (MSD) of cells was calculated using the following equation:

$$\text{MSD}=\langle r^{2}(\tau =i\Delta t)\rangle =\sum_{k=1}^{n-i}\frac{\left\{{\left(x_{k+i}-x_{k}\right)}^{2}+{\left(y_{k+i}-y_{k}\right)}^{2}\right\}}{n-i}$$ (1)

where r is the displacement travelled by a cell during time interval τ, i is the number of frames, Δt is unit time, *x*_*k*_ and *y*_*k*_ are cell coordinates at *k* frames, and n is the total number of frames. MSD is proportional to the power of τ as follows:

$$\text{MSD}\propto \tau^{\alpha }$$ (2)

where the exponent α indicates the degree of randomness. If cell movement is a random walk, α is equal to 1. The log–log plot of τ and MSD were fitted with the following equation:

logf(i)=αlogτ+log(4D) (3)

where α and D are the fitting parameters and D is a nominal diffusion constant. Randomness of cell movement can be determined from α[23].

Migration speed *v* was evaluated by measuring migration distance traveled between two sequential frames divided by the time interval of the frames (15 min). Speed distribution was fitted by the following equation based on the two dimensional Maxwell-Boltzmann speed distribution:

$$f(v)=av\text{exp}(-bv^{2})$$ (4)

where *v* is speed and *a* and *b* are the fitting parameters.

### Simulation of two-dimensional random walk

Each particle started from the original point (0, 0) and randomly moved, with equal probability and equal step size, in one of four directions (1, 1), (1, -1), (-1, 1), and (-1, -1).

## Results

### Activation of Activin/Nodal and Wnt/beta-catenin pathway induced mesendodermal differentiation and EMT

To confirm that hiPSCs differentiated into mesendodermal cells during gastrulation, we first assessed the expression of mesendodermal and EMT markers. Activin A, an Activin/Nodal activator protein, and CHIR, a small-molecule activator of Wnt/β-catenin signaling, were used to induce mesendodermal differentiation [12]. CHIR is also known to upregulate nodal pathway and p-smad2 levels as well as β-catenin levels [24]. Cells were maintained in the undifferentiation-maintaining medium containing basic fibroblast growth factor (bFGF) and a low concentration of activin. The medium was replaced with fresh undifferentiation-maintaining medium or differentiation induction medium containing high concentrations of activin and CHIR, and the cells were incubated for 2 days (Fig 1b and S1 Table). We assessed the expression of four marker proteins, namely, OCT3/4 and E-cadherin, (markers of undifferentiated hiPSCs), BRACHYURY (a marker of mesendodermal differentiation) and SNAIL (a marker of EMT) [25, 26].

**Fig 2:**
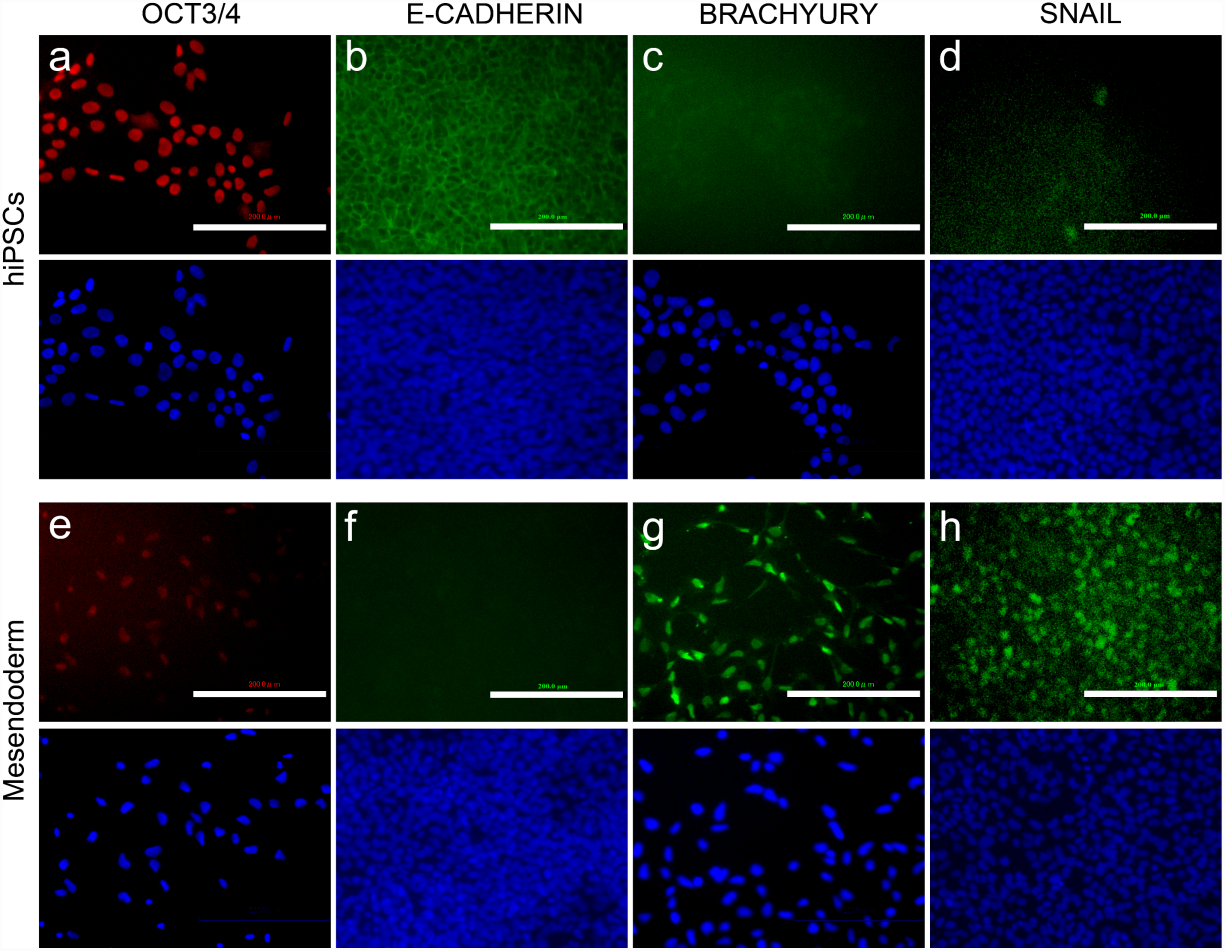
hiPSC-derived cells expressed mesendodermal and EMT markers. Fluorescent images from immunostaining analysis at 2 days after differentiation using anti-OCT3/4 (a and e), anti-E-cadherin (b and f), anti- BRACHYURY (c and g), and anti-SNAIL antibodies (d and h). a–d: Undifferentiated cells. e–h: Differentiated mesendodermal cells derived from hiPSCs by activating the Activin/Nodal and Wnt/beta-catenin pathways. Blue: DAPI. Scale bars are 200 μm.

E-cadherin expression is downregulated during EMT, and BRACHYURY is expressed in the primitive streak, which allows cell migration during gastrulation. Immunocytochemical analysis showed that undifferentiated cells expressed OCT3/4 and E-cadherin but did not express BRACHYURY and SNAIL (Fig 2a–2d) and that differentiated cells expressed BRACHYURY and SNAIL but did not express OCT3/4 and E-cadherin (Fig 2e–2h). Downregulation of E-cadherin, the main cell-cell adhesion molecule of undifferentiated hiPSCs [25], was also confirmed by the detachment of cells following mesendodermal differentiation. Although the undifferentiated hiPSCs attached to each other to form two-dimensional cell aggregates (Fig 2a–2d), mesendodermal differentiation caused cell detachment and allowed individual cells to be isolated (Fig 2e–2h). These results suggest that activin and CHIR successfully induced mesendodermal differentiation and EMT, which occur during gastrulation *in vivo*.

### Mesendodermal differentiation increases migration speed

The hiPSCs were dissociated into single cells before plating them on a dish to reduce cell-cell interaction (time 0 h in Fig 1b). Time-lapse imaging of undifferentiated cells and mesendodermal cells was performed at 15-min intervals to determine changes in cell motility during gastrulation (Fig 1b). Given that EMT confers migratory and invasive properties to cells during gastrulation [2, 4], differentiated mesendodermal cells may show increased migration.

**Fig 3:**
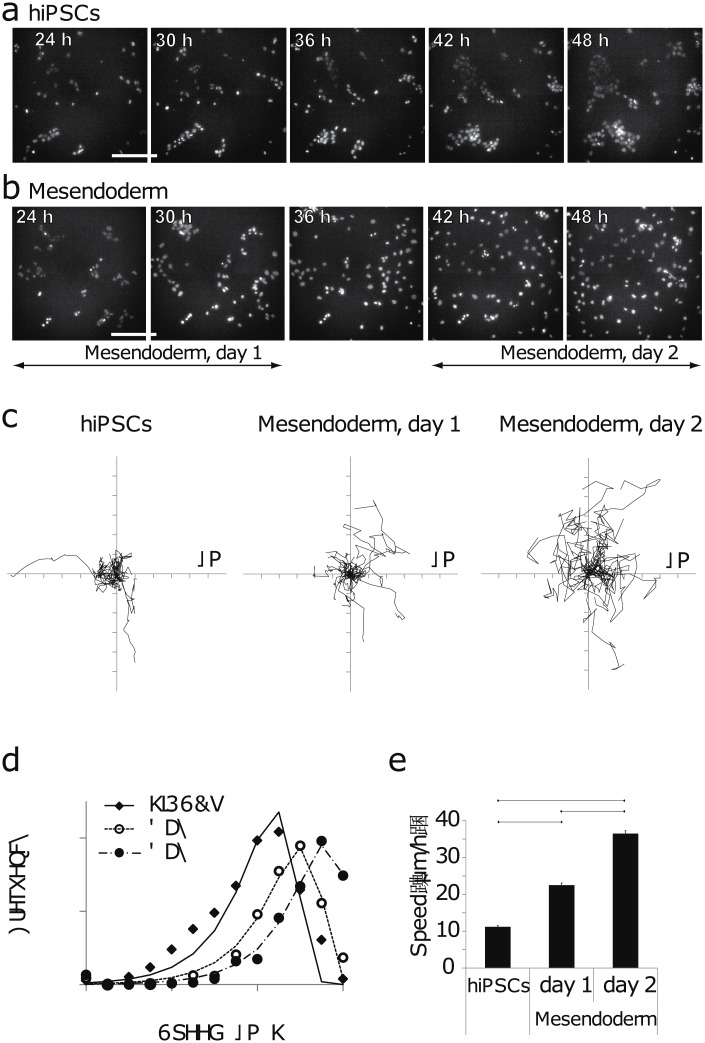
Time-lapse imaging analysis demonstrates that mesendodermal differentiation increases migration speed. a and b: Fluorescent images of undifferentiated hiPSCs (a) and differentiated mesendodermal cells (b). Scale bars are 100 μm. c: Trajectories of twenty cells were overlapped by setting their starting positions at the origin (0, 0). d and e: Distribution (d) and average (e) of cell migration speed. (d) The marks are measured data and the lines are the fitting lines based on Eq 4. The square of the correlation coefficient (*r*^2^) between the measured data and the fitting lines was 0.94 (hiPSCs), 0.99 (day 1), and 0.98 (day 2), respectively. (e) One-way ANOVA P < 2 × 10^−16^. Mean ± SE. n = 1440, 1440, 957. * P < 0.05, *** P < 0.001 (Welch *t*-test with Bonferroni’s correction).

Time-lapse imaging showed that undifferentiated hiPSCs moved slowly and that a portion of the cells assembled to form loose colonies (Fig 3a and S1 Movie), whereas differentiated mesendodermal cells dissociated from each other and showed a gradual increase in their migration speed (Fig 3b and S2 Movie), suggesting the occurrence of EMT. To quantitatively analyze cell movement, we tracked the position of nuclei stained with the live cell nuclear dye, Hoechst 33342. We defined 24–30 h and 42–48 h of mesendodermal differentiation as day 1 and day 2, respectively (Figs 1b and 3b). Fig 3 shows the trajectory of 20 cells, which are overlapped by setting the starting point to the origin. The trajectories of undifferentiated cells were dense around the origin, suggesting that those cells moved slowly and/or were trapped around the origin (Fig 3c). In contrast, the trajectories of mesendodermal cells were sparse especially at day 2, suggesting that these cells moved faster and/or straighter (more ballistically) (Fig 3c). The migration speed under these three conditions showed a single-peak distribution and not a double-peak distribution (Fig 3d). Moreover, the peak shifted to the right and average migration speed increased after mesendodermal differentiation (Fig 3d and 3e). Cell densities at the initial frame of undifferentiated hiPSCs and mesendodermal differentiation on day 1 and day 2 were about 2%, 2%, and 7% of the close-packed cell density (calculated as 4.5 × 10^5^ cells/cm^2^) respectively, suggesting that cell-cell interactions were low. The increase in cell density from day 1 to day 2 was mainly due to cell influx from outside the frame. These observations are consistent with the immunocytochemical results, and indicate the occurrence of EMT, as characterized by the degradation of E-cadherin-mediated cell–cell adhesions which promote cell dissociation and migration. This suggests the efficacy of our system as a model for analyzing the dynamics of mesendodermal cells that occur during gastrulation *in vivo*.

### Random walk analysis indicating that both hiPSCs and differentiated mesendodermal cells migrate randomly

Next, we analyzed the randomness or linearity of cell movement. To assess randomness of cell movement, random walk analysis, which calculates MSD as a function of a time interval (τ), was performed and plotted on a log–log graph (Eq 1). Randomness was determined from the slope (α) of the log–log graph of MSD and τ (Eqs 2 and 3). For random or linear cell movement, α is equal to 1 or 2, respectively. If cells movement is ballistic, their trajectories are straighter than those of cells showing normal random walk behavior (2 > α > 1). For confined motion such as random walk in an infinite valley, α is <1.

**Fig 4:**
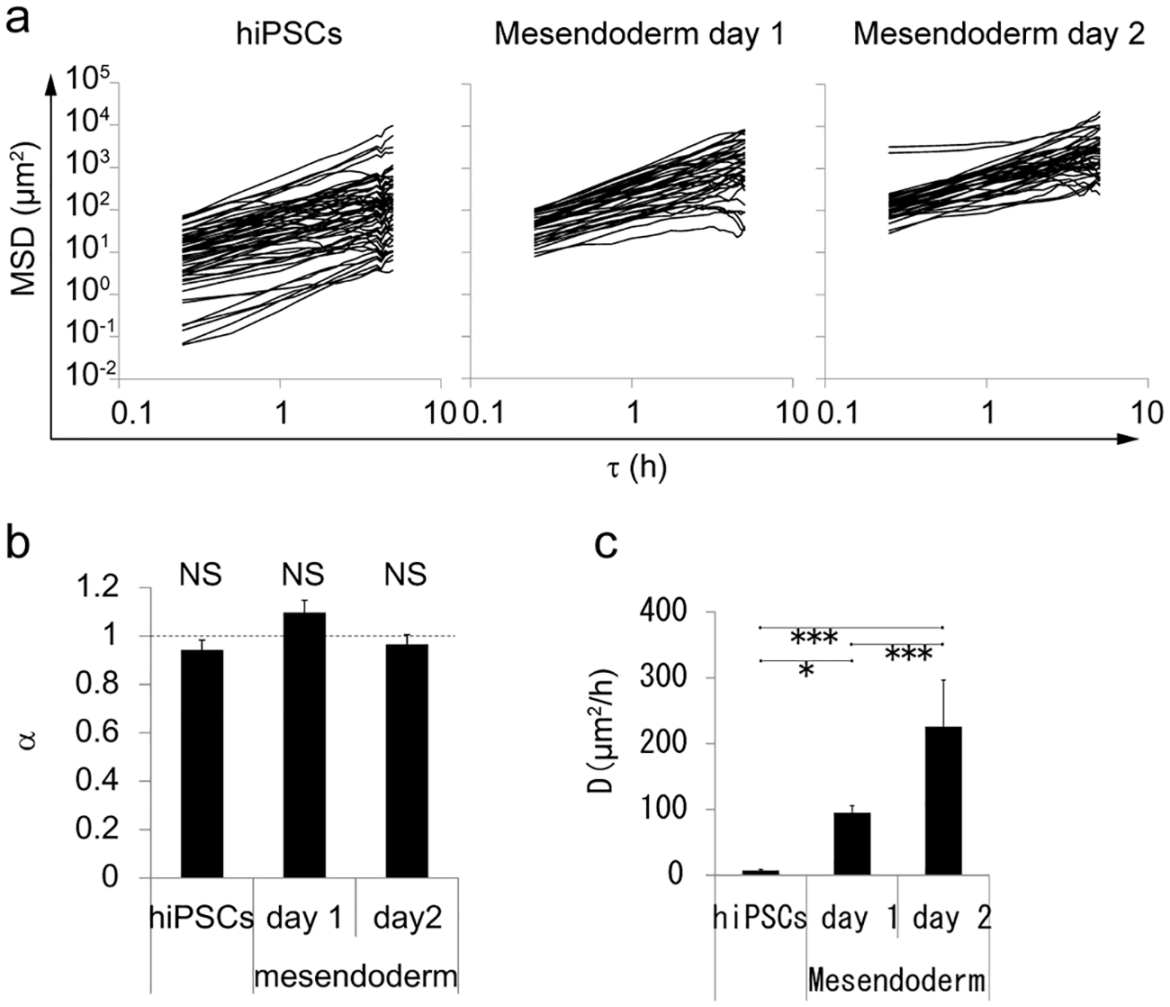
Random walk analysis shows that both hiPSCs and differentiated mesendodermal cells migrate randomly. Random walk analysis based on Eq 3. MSDs of 20 cells were plotted against time intervals (*τ*) in a log–log plot (**a**). The average of the slopes (**b**, α value in Eq 3). NS Non-significant (P > 0.05, one sample *t*-test; alternative hypothesis: true mean is not equal to 1). The average of the nominal diffusion constant calculated from the intercept (**c**, D in Eq 2)., Mean ± SE; n = 60 for undifferentiated cells and 40 for mesendodermal cells. One-way ANOVA P <1 × 10^−7^. * P < 0.05, *** P < 0.001 (Welch *t*-test with Bonferroni’s correction).

The results showed that almost all slopes approximated 1, in all conditions (Fig 4b and 4c). The average α values (mean ± SE) for undifferentiated hiPSCs on day 1, differentiated mesendodermal cells on day 1, and differentiated mesendodermal cells on day 2, were 0.94 ± 0.4, 1.10 ± 0.05, and 0.96 ± 0.04, respectively (Fig 4c). The analysis indicated that the average α value was not statistically different from 1 in the three conditions. We also estimated the nominal diffusion constant of cell movement, D (Eq 3), which increased with mesendoderm differentiation. The correlation coefficient of the diffusion constant and the migration speed was 0.922, suggesting the increase in the diffusion constant could be explained by the increase in migration speed. We also fitted the speed distribution based on the two dimensional Maxwell-Boltzmann speed distribution, which also assumed random motion (Eq 4, Fig 3d). Measured data and fitted values agreed well. These results indicate that both hiPSCs and differentiated mesendodermal cells migrate randomly.

## Discussion

In the present study, we constructed a single-cell observation system, *in vitro*, to assess the migration of mesendodermal cells, which correspond to that of the human gastrulation stage. Our findings are the first to indicate that both hiPSCs and differentiated mesendodermal cells migrated randomly.

Immunostaining analysis illustrated the differentiation of hiPSCs into mesendodermal cells, and occurrence of EMT. Time-lapse imaging indicated that mesendodermal cells underwent dissociation and showed increased migration speed. Combined, our results suggest that mesendodermal cells correspond to those undergoing EMT during gastrulation. EMT occurs widely in the body, performing its natural functions during embryo formation, wound healing, and tumor metastasis [27]. However, the mechanisms of EMT are not well known. Since we used a monolayer culture in a defined medium, our system might be a good model for observing and controlling the dynamics of wound healing and tumor metastasis as well as human embryonic development, which have not been well studied, because of ethical and technical limitations. In our experiment, ROCK inhibitor was used to suppress dissociation induced apoptosis [16]. ROCK inhibition has some effect on differentiation and migration [28]. However, as EMT was observed in this experiment, the ROCK inhibition effect may be weak in EMT. As such, it may be necessary to analyze the effects of other reagents.

Undifferentiated hiPSCs, which correspond to epiblast cells, move randomly as seen in mesendodermal cells. However, they move more slowly than mesendodermal cells. Because epiblast cells are epithelial cells, their slow migration can be expected. As undifferentiated hiPSCs adhere to each other by E-cadherin-mediated cell-cell adhesion, a confined random walk where α < 1 was expected. However, a confined random walk was not observed in the present study. Because the cells were plated after dissociation into single cells at low density (less than 7% of close-packed density), cell-cell adhesions might be insufficient to anchor the cells. Thus, the possibility that the cells may have migrated independently needs to be considered. Therefore, additional studies may be needed to determine dependency of randomness (α) on cell density. Moreover, there is a possibility that complex motion went unnoticed. Because cell signaling and cell-cell interaction was not completely controlled in this experiment, complex motion may be contained in randomness. Detailed studies may be needed in the future to address such issues.

**Fig 5:**
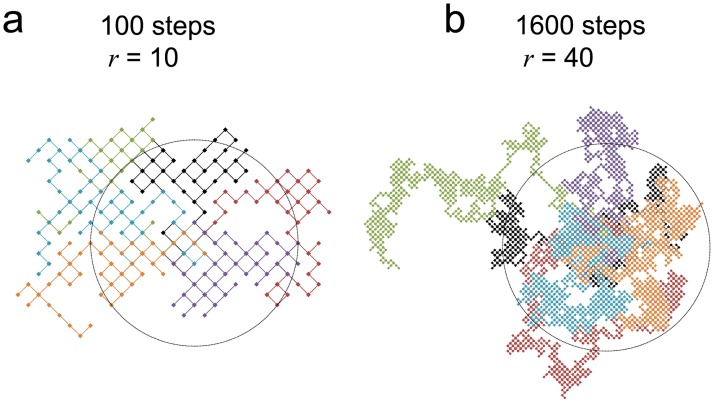
Two-dimensional random walk simulation demonstrates that a particle can reach all parts during the random walk. Simulation of two-dimensional random walk at 100 (a) and 1600 steps (b). Trajectories and locus points of 6 particles are shown in the different colors. Particles started from the center of the circle and moved randomly in four directions. The radiuses (*r*) of the circles are the square root of the steps (*n*). Note that *n* and *r* in b are 16-times and 4-times larger than those in a, respectively.

Mesendodermal cells also migrated randomly. In mouse mesoderm and zebra fish endoderm cells that form the intestine also migrate randomly in vivo [29, 30]. These findings are consistent with our results. Human endodermal cells migrate from the primitive streak to form a discoidal layer (Fig 1a) [2, 10]. The theory of a two-dimensional random walk instead of a one- or three-dimensional random walk suggests that the probability to reach a point is 1 [31, 32]. Such a probability may also be explained from a simplified viewpoint as follows. In the case of random walk, the MSD is proportional to the time interval (MSD ∝ *τ* = *n*Δ*t*). In the two-dimensional case, the average area covered by total steps (*n*) is proportional to the number of steps (〈*πr*^2^ (*n*Δ*t*)〉 = *πMSD* ∝ *n*, Fig 5b). In the area, there are *n* locus points, and therefore the density of locus points is independent of step number ($\frac{\langle {\pi r}^{2}(n\Delta t)\rangle }{n}=constant$), suggesting that a particle can reach all points during the random walk (Fig 5c). These results suggest a possibility that random movement is suitable to effectively disperse cells homogeneously from the primitive streak to form the discoidal layer of the endoderm. These considerations may lead to the hypothesis that, during gastrulation, germ layers form in a self-organized manner without programmed step-by-step instructions. To clarify these possibilities, further studies are required using experimental conditions that closely resemble *in vivo* conditions, including separate induction of the mesoderm and endoderm, high cell-density culturing and chemical cues for chemotaxis. However, the findings of the present study may serve as a basis for further studies aimed at determining the dynamics of human embryogenesis.

## Conclusion

**Fig 6:**
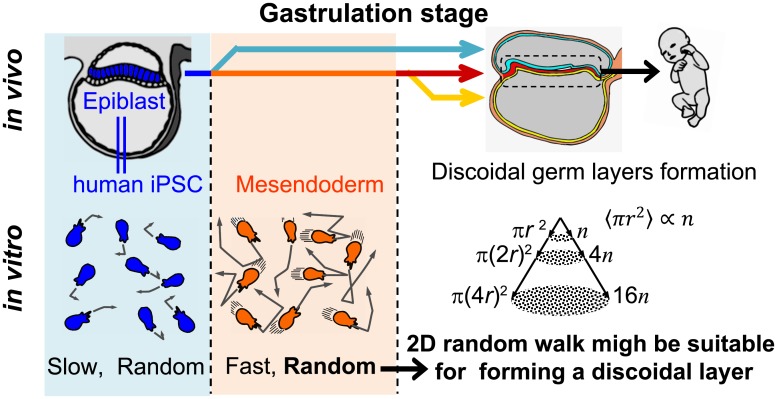
Schematics of this study.

Our study utilized hiPSCs to mimic human gastrulation dynamics *in vitro* (Fig 1). Human iPSC-derived mesendodermal cells expressed specific marker proteins (Fig 2) and the migration speed was increased (Fig 3). These results suggest that we successfully induced mesendodermal differentiation and EMT, which occur during gastrulation, *in vivo*. Mesendodermal cells migrated individually and rapidly as expected, but randomly (Fig 4). A two-dimensional random walk is suitable for dissociating particles homogeneously to form a discoidal layer (Fig 5). Thus, random migration may aid the formation of discoidal layers during gastrulation (Fig 6).

## Supporting information

S1 Movie Time lapse imaging of undifferentiated hiPSCs. Nuclei were stained with Hoechst33342. 15 min/frame. From 24 to 48 hours after plating.
(GIF)

S2 Movie Time lapse imaging of mesendodermal cells derived from hiPSCs. Nuclei were stained with Hoechst33342. 15 min/frame. From 24 to 48 hours after plating.
(GIF)

S1 Table Composition of the defined culture media. (DOCX)

S2 Table Anti-bodies used. (DOCX)
