# Supplementary figures and images for: Random migration of induced pluripotent stem cell-derived human gastrulation-stage mesendoderm
Source: PLoS One. 2018 Sep 10;13(9):e0201960. doi: 10.1371/journal.pone.0201960 (PMC6130871; doi:10.1371/journal.pone.0201960)

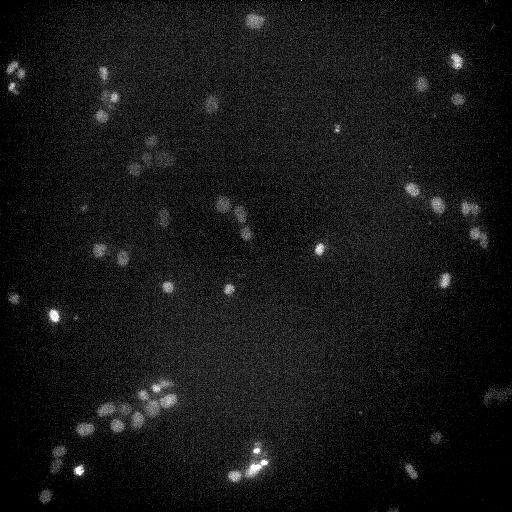

Supplement: S1 Movie — Nuclei were stained with Hoechst33342. 15 min/frame. From 24 to 48 hours after plating. (GIF) [file pone.0201960.s001.gif]

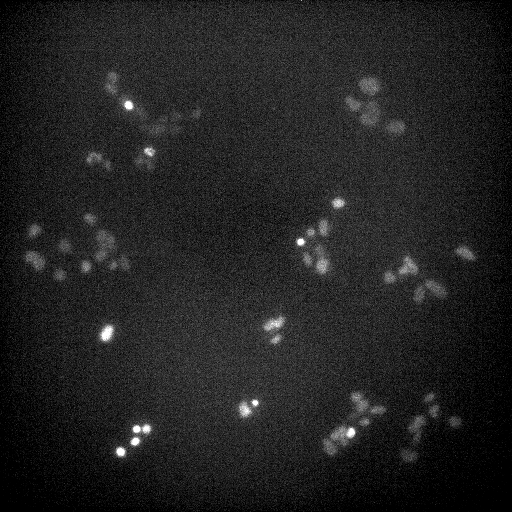

Supplement: S2 Movie — Nuclei were stained with Hoechst33342. 15 min/frame. From 24 to 48 hours after plating. (GIF) [file pone.0201960.s002.gif]
