# Supplementary material for: Random migration of induced pluripotent stem cell-derived human gastrulation-stage mesendoderm
Source: PLoS One. 2018 Sep 10;13(9):e0201960. doi: 10.1371/journal.pone.0201960 (PMC6130871; doi:10.1371/journal.pone.0201960)
Supplement: S1 Table — (DOCX) [file pone.0201960.s003.docx]

## S1 Table. Composition of the defined culture mediums

|  | Undifferentiation  -maintaining medium (hESF-9a^a^) | Mesendoderm  -induction medium*^b^* | |
| --- | --- | --- | --- |
| hESF-8 medium |  | | |
| Basal medium | hESF-Grow*^c^* | | |
| Bovine pancreas insulin (I-5500*^d^*) | 10 μg/mL | | |
| Human apotransferrin (T-1147*^d^*) | 5 μg/mL | | |
| 2-Mercaptoethanol (M-7522*^d^*) | 10 μM | | |
| Ethanolamine (E-0135*^d^*) | 10 μM | | |
| Sodium selenite (S-9133*^d^*) | 20 nM | | |
| L-ascorbic acid-2-phosphate (013-196411*^e^*) | 100 ng/mL | | |
| Oleic acid*^f^* (O-3008*^d^*) | 4.7 μg/mL | | |
| Heparin sodium salt from (H-3149*^d^*) | 100 ng/mL | | |
| Supplements |  | | |
| Basic fibroblast growth factor (bFGF) *^g^* | 10 ng/mL | |  |
| Human recombinant activin A (338-AC*^h^*) | 2 ng/mL (low) | | 10 ng/mL (high) |
| CHIR99402 (CHIR, 039-20831*^e^* ) |  | | 12 μM |

*^a^* Yoshimitsu, R., K. Hattori, S. Sugiura, Y. Kondo, R. Yamada, S. Tachikawa, T. Satoh, A. Kurisaki, K. Ohnuma, M. Asashima, and T. Kanamori. 2014. Microfluidic perfusion culture of human induced pluripotent stem cells under fully defined culture conditions. Biotechnol. Bioeng. 111:937-947.

*^b^* Ninomiya, H., K. Mizuno, R. Terada, T. Miura, K. Ohnuma, S. Takahashi, M. Asashima, and T. Michiue. 2015. Improved efficiency of definitive endoderm induction from human induced pluripotent stem cells in feeder and serum-free culture system. In Vitro Cellular & Developmental Biology-Animal 51:1-8

*^c^* Obtained from Cell Science & Technology Institute, Miyagi, Japan

*^d^* Sigma Aldrich, St. Louis, MO, USA

*^e^* Wako Pure Chemical Inc., Osaka Japan

*^f^* Conjugated with 0.5 mg/mL of fraction V fatty acid-free bovine serum albumin

*^g^* Katayama Science, Japan

*^h^* R&D Systems, Minneapolis, MN, USA
