# Supplementary material for: Random migration of induced pluripotent stem cell-derived human gastrulation-stage mesendoderm
Source: PLoS One. 2018 Sep 10;13(9):e0201960. doi: 10.1371/journal.pone.0201960 (PMC6130871; doi:10.1371/journal.pone.0201960)
Supplement: S2 Table — (DOCX) [file pone.0201960.s004.docx]

## Supplementary Table 2. Anti-body used

|  | 1^st^ antibody | 2^nd^ antibody |
| --- | --- | --- |
| OCT3/4 | anti-human Oct3/4(H-134) rabbit polyclonal IgG, sc-9081*^a^* | goat anti-rabbit IgG(H+L) Alexa Fluor 546, A11035*^b^* |
| E-cadherin | anti-human E-cadherin (67A4), mouse monoclonal, IgG_1_ kappa light chain, sc21791*^a^* | goat anti-mouse IgG_1_ Alexa Fluor 488, A21121*^b^* |
| T | anti-human Brachyury, goat polyclonal IgG, AF2085*^c^* | rabbit anti-goat IgG(H+L) Alexa Fluor 488 A21222*^b^* |
| SNAL | anti-human SNAIL Antibody, goat polyclonal IgG, AF3639*^c^* | rabbit anti-goat IgG(H+L) Alexa Fluor 488 A21222*^b^* |

*^a^* SantaCruz Biotechnology, CA, USA

*^b^* Life Technologies, California, USA

*^c^* R&D systems Inc., Minneapolis, MN, USA
